# The Beat

**DOI:** 10.1289/ehp.119-a338b

**Published:** 2011-08-01

**Authors:** Erin E. Dooley

## EPA Rule Targets Smokestack Pollution

In July 2011 the U.S. EPA finalized the Cross-State Air Pollution Rule to reduce SO_2_ and NO_X_ emissions from power plants using available emissions control technologies.^1^ SO_2_ and NO_X_ contribute to ground-level ozone and fine particle pollution and often cross state lines, ending up far from their provenance. The rule is projected to achieve up to $280 billion in annual health benefits by preventing avoidable illnesses, sick days at work, and premature deaths. Within three years the rule is expected to help reduce SO_2_ emissions by 73% and NO_X_ emissions by 54% over 2005 levels.

## Extremotolerant Fungi Isolated in Dishwashers

Dishwasher interiors are extreme environments featuring high temperatures, high pH, and high salinity, supplemented by high organic content from the dirty dishes. A study of households on six continents has found several species of potentially pathogenic “extremotolerant” fungi growing in the rubber seals on household dishwashers.^2^ The most commonly isolated fungi were *Exophiala* species, which can colonize the lungs of patients with cystic fibrosis and occasionally cause fatal infections in healthy humans; these were found in over half the dishwashers sampled.

## Japan Struggles with Debris from Tohoku Disaster

The Japanese prefectures affected by the March 2011 Tohoku earthquake and tsunami are struggling to recover, but the infrastructure for disposing of an estimated 21.83 million tons of debris is not yet available. According to Japanese news service Asahi Shimbun, some 35% of the disaster debris has been moved to temporary dump sites in an effort to clear space for rebuilding.^3^ The land around Ishinomaki Commercial High School in Miyagi Prefecture is the site of a dusty, foul-smelling dump for more than 100,000 tons of concrete, wood, household goods, and tsunami sludge contaminated with unquantified chemical agents.^4^ Students and staff at the school are reporting respiratory effects and eye irritation they attribute to the dump.^3^

## NIH Funds Oil Spill Health Effects Study

In July 2011 the NIH announced a new five-year, $25.2-million project to conduct research on health effects of the BP *Deepwater Horizon* disaster of 2010.^5^ Led by the NIEHS, the project will support population-based and laboratory studies at four Gulf Coast universities. The universities will partner with community organizations to incorporate local concerns into study designs and communicate findings of the research. Each university also will implement a community resilience program to study factors that affect how well individuals, households, and communities adapt to events such as the *Deepwater Horizon* disaster. The findings will be used to help improve capacity to respond to future disasters.

## House Passes Amendment to Block Approval of GE Salmon

The U.S. House of Representatives has passed an amendment to the Fiscal Year 2012 Appropriations bill for the FDA^6^ that would prohibit the agency from using federal funds to approve AquaBounty^®^ salmon, a genetically engineered fish designed to grow faster than conventional salmon.^7^ The amendment was introduced by Representative Don Young (R–AK), who argued the AquaBounty salmon could threaten wild salmon populations and lower the price of their wild counterparts. Other supporters cited the lack of long-term studies on the safety of eating genetically engineered fish. The bill now awaits action by the Senate Appropriations Committee.
